# Encapsulation of Citrus By-Product Extracts by Spray-Drying and Freeze-Drying Using Combinations of Maltodextrin with Soybean Protein and ι-Carrageenan

**DOI:** 10.3390/foods7070115

**Published:** 2018-07-19

**Authors:** Konstantinos Papoutsis, John B. Golding, Quan Vuong, Penta Pristijono, Costas E. Stathopoulos, Christopher J. Scarlett, Michael Bowyer

**Affiliations:** 1 School of Environmental and Life Sciences, The University of Newcastle, P.O. Box 127, Ourimbah, NSW 2258, Australia; john.golding@dpi.nsw.gov.au (J.B.G.); vanquan.vuong@newcastle.edu.au (Q.V.); penta.pristijono@newcastle.edu.au (P.P.); c.scarlett@newcastle.edu.au (C.J.S.); michael.bowyer@newcastle.edu.au (M.B.); 2 NSW Department of Primary Industries, Locked Bag 26, Gosford, NSW 2250, Australia; 3 Division of Food and Drink, School of Science, Engineering and Technology, University of Abertay, Dundee DD1 1HG, UK; c.stathopoulos@abertay.ac.uk

**Keywords:** citrus by-products, encapsulation, phenolic compounds, antioxidant capacity, polysaccharides, protein

## Abstract

The effect of different combinations of maltodextrin (MD) coating agents (MD, MD + soybean protein, and MD + ι-carrageenan) on the encapsulation of lemon by-product aqueous extracts using freeze-drying and spray-drying were investigated. The total phenolic content (TPC), total flavonoid content (TFC), and ferric ion reducing antioxidant power (FRAP) of the microparticles were evaluated. Freeze-drying with the mixture of MD + soybean protein resulted in the highest retention of TPC, TFC, and FRAP (1.66 ± 0.02 mg GAE/g d.b., 0.43 ± 0.02 mg CE/g d.b., and 3.70 ± 0.05 mM TE/g, respectively). Freeze-drying resulted in microparticles with lower moisture content (MC) and water activity (a_w_) than those produced by spray-drying. Specifically, the MC and a_w_ of the microparticles produced by freeze-drying ranged from 1.15 to 2.15% and 0.13 to 0.14, respectively, while the MC and a_w_ of the microparticles produced by spray-drying ranged from 6.06% to 6.60% and 0.33 to 0.40, respectively. Scanning electron microscopy revealed that spray-drying resulted in the formation of spherical particles of different sizes regardless of the type of coating agent. Although freeze-drying resulted in microparticles with amorphous glassy shapes, the mixture of MD + soybean protein resulted in the formation of spherical porous particles. X-ray diffraction revealed a low degree of crystallinity for the samples produced by both techniques.

## 1. Introduction

Huge amounts of citrus by-products are generated annually by the juice industry [1]. These residues are a significant potential source of polyphenols, which possess radical scavenging properties towards oxygen species, and complexing properties towards proteins [2]. Due to the presence of unsaturated bonds in their molecular structure, polyphenols are susceptible to light, heat, and oxygen degradation [1,3]. Therefore, extracts enriched in polyphenols should be treated appropriately in order to produce a product that is able to maintain its integrity.

Encapsulation is employed to protect chemically sensitive bioactive compounds from degradation due to adverse environmental conditions and is also used to control the release of the encapsulate [4]. Among the different techniques used for the encapsulation of bioactive compounds, spray-drying is widely used in the food industry due to its rapidity and low cost [5]. However, spray-drying conditions for the encapsulation of polyphenols must be optimized in order to avoid accelerated degradation. Freeze-drying is another technique which can be used for the encapsulation of bioactive compounds, especially for those that are susceptible to degradation at high temperatures [6]. Compared with spray-drying, freeze-drying suffers from comparative disadvantages including significantly higher processing times and higher unit cost. 

Several studies have shown that the encapsulation efficiency of polyphenols and the physical properties of the microparticles may be affected by the encapsulation methods employed, as well as by the type of the coating agent [7,8]. For instance, Ballesteros et al. [6] compared the retention of antioxidant phenolic compounds extracted from coffee grounds and encapsulated by freeze-drying and spray-drying using different coating agents. The authors found that both the encapsulation technique and the coating agent affected the retention of antioxidants, as well as the morphology of the microparticles. Therefore, in order to maximize the retention of polyphenols within the microparticles, as well as to minimize the operation cost, both the coating agent and the encapsulation method should be thoroughly assessed.

A range of coating agents can be utilized for the encapsulation of polyphenols, including maltodextrin, gum arabic, polydextrose, and proteins [6,8,9]. Maltodextrins are D-glucose polymers that are usually used for the encapsulation of polyphenols due to their high solubility, low viscosity, and good gel formation properties [10,11]. Proteins have also been considered as potential coating agents due to their film forming properties and their favorable interactions with polyphenols [12]. Carrageenans are sulfated polysaccharides extracted from algae and have been extensively used in the food industry as gelling agents, stabilizers and texture enhancers [13]. ι-Carrageenan has been reported as a promising film-forming material but has not been extensively studied as a coating agent in microencapsulation [14]. Table 1 summarizes the different coating agents that have been used for the encapsulation of citrus by-product extracts. 

It has been recently mentioned that the mixture of coating agents might be more efficient than the individual compounds for the encapsulation of polyphenols [15]. Kuck and Noreña [8] reported that a mixture of 5% partially hydrolyzed gum with 5% polydextrose was more efficient coating agent than the individual ones during spray-drying for the encapsulation of grape skin phenolic compounds. The aim of this study was the encapsulation of lemon by-product aqueous extracts by two encapsulation techniques (spray-drying and freeze-drying), using combinations of maltodextrin with soybean protein and ι-carrageenan, as coating agents.

## 2. Materials and Methods

### 2.1. Materials

Lemon waste (including peels, membranes, and seeds) was kindly provided by Eastcoast Food and Beverages, Kulnura, NSW, Australia. The seeds were thoroughly removed from the waste and the remaining peels and membranes (lemon by-product) were stored at −18 °C until use. Lemon by-products were dried by freeze-drying (FD3 freeze dryer; Thomas Australia Pty. Ltd., Seven Hills, NSW, Australia) as described by Papoutsis et al. [22]. The dried material was ground using a commercial blender (Waring 2-speed blender, John Morris Scientific, Chatswood, Australia), and then sieved through a 1.40 mm steel mesh sieve prior to extraction, as this particle size has been previously shown to result in the highest recovery of bioactive compounds and antioxidants from citrus waste [23]. The dried, sieved powder was stored at −18 °C for further analysis.

### 2.2. Extraction Process

The extraction of polyphenols and antioxidants (core material) from the lemon by-products was conducted using the optimized hot water extraction conditions reported by Papoutsis et al. [24]. Five grams of lemon by-product powder was mixed with 100 mL of water and placed in a water bath (Labec Laboratory Equipment Pty. Ltd., Marrickville, NSW, Australia) at 95 °C for 15 min. After extraction, the aqueous extracts were vacuum-filtered using a Whatman no.1 filter paper and stored at 4 °C until use.

### 2.3. Encapsulation of the Extracts

**Figure 1:**
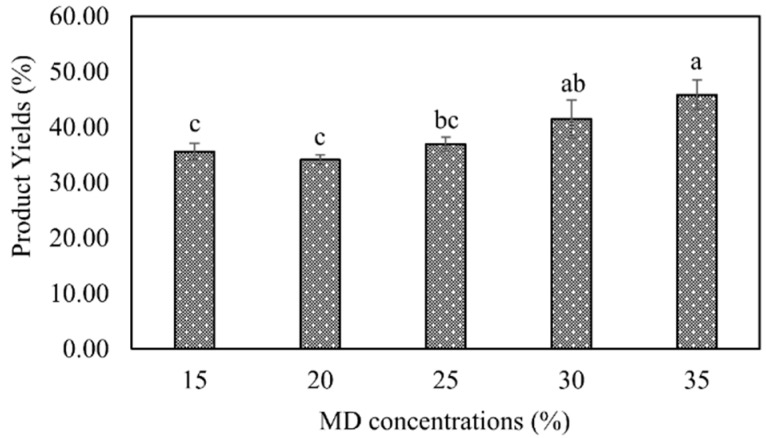
Product yields using different maltodextrin (MD) concentrations into lemon by-product aqueous extracts during spray-drying; Bars followed by different letters are significantly different at *P* < 0.05, according to the Duncan’s test.

**Figure 2:**
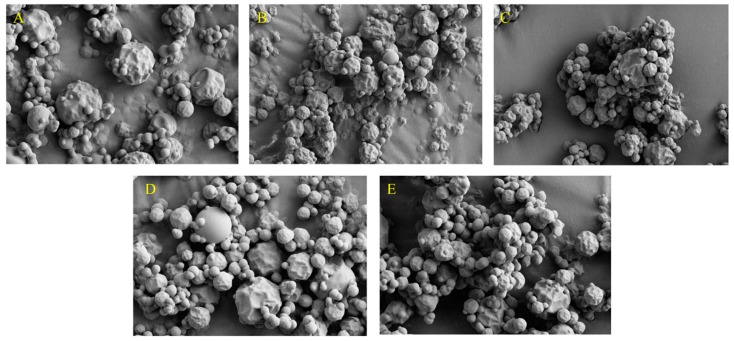
Scanning electron micrographs of spray dried microparticles: (**A**) 15% Maltodextrin, (**B**) 20% Maltodextrin, (**C**) 25% Maltodextrin, (**D**) 30% Maltodextrin, (**E**) 35% Maltodextrin.

For the encapsulation of lemon by-product aqueous extracts, two techniques (spray-drying and freeze-drying) were employed and compared. Maltodextrin (16.5–19.0 dextrose equivalent (DE)) and its mixtures with soybean protein isolate or ι-carrageenan were investigated as coating agents (Table 2). The coating agent was mixed with the lemon by-product aqueous extracts in a concentration of 30% (*w*/*v*). The concentration of coating agent in the lemon by-product aqueous extracts was selected based on preliminary experiments, in which five concentrations (15, 20, 25, 30, and 35% *w*/*v*) were tested using maltodextrin of 16.5–19.0 DE as the coating agent and spray-drying as the encapsulation technique, considering the product yields (Figure 1) and the morphology of the microparticles (Figure 2). The concentrations of 30 and 35% (*w*/*v*) maltodextrin resulted in spherical uniform microparticles, with less concavities and smoother surfaces (Figure 2). Microparticles with rough surfaces are more susceptible to oxidation since they have larger contact areas than the smooth ones [25]. Spray-drying was performed using a Buchi mini spray dryer B-290 (Noble Park, VIC, Australia) at the following conditions: Inlet temperature of 125 °C; maximum outlet temperature of 55 °C; atomization air flow rate of 601 L/h; liquid feed pump rate of 4 mL/min; main drying air flow rate of 38 m^3^/h; feed solution temperature 70 °C and feed solution of 70 mL [20]. In the freeze-drying process, the samples were initially frozen using liquid nitrogen and then freeze dried for 48 h (FD3 freeze dryer, Thomas Australia Pty. Ltd., Seven Hills, NSW, Australia).

### 2.4. Sample Characterization

#### 2.4.1. Total Phenolic Content (TPC)

The TPC of the microparticles was determined as described by Škerget et al. [26]. 2.5 mL of 10% (*v*/*v*) Folin–Ciocalteu reagent was mixed with a 0.5 mL sample, followed by the addition of 2 mL of 7.5% (*w*/*v*) Na_2_CO_3_. The mixture was incubated for 1 h at ambient temperature, before the absorbance was recorded at 760 nm. The results were calculated using a standard curve (R^2^ = 0.9923) which was built by dissolving gallic acid in water at different concentrations (0, 12.5, 25, 50, 80, and 100 µg/ mL) and expressed as mg of gallic acid equivalents per g sample on dry basis (mg GAE/g d.b.).

#### 2.4.2. Total Flavonoid Content (TFC)

The TFC of the microparticles was determined according to Zhishen et al. [27]. 2 mL of H_2_O were mixed with 0.15 mL of 5% (*w*/*v*) NaNO_2_ and a 0.5 mL sample and incubated at ambient temperature for 6 min. Then, 0.15 mL of 10% (*w*/*v*) AlCl_3_ was added and the mixture was left at room temperature for 6 min. Subsequently, 2 mL of 4% (*w*/*v*) NaOH and 0.7 mL of H_2_O were added and the solution left at room temperature for 15 min before the absorbance was measured at 510 nm. The results were calculated by using a standard curve (R^2^ = 0.9928) which was built by dissolving catechin in methanol at different concentrations (0, 12.5, 25, 50, 80, and 100 µg/ mL) and expressed as mg of catechin equivalents per g sample on dry basis (mg CE/g d.b.).

#### 2.4.3. Ferric Ion Reducing Antioxidant Power (FRAP)

The FRAP of the microparticles was measured according to Thaipong et al. [28]. A working FRAP solution was prepared by mixing 300 mM acetate buffer, 10 mM TPTZ (2,4,6-tripyridyl-s-triazine) in 40 mM HCl and 20 mM FeCl_3_ in the ratio of 10:1:1 and warmed at 37 °C. A 0.15 mL of sample was mixed with 2.85 mL of working FRAP solution and incubated at room temperature for 30 min in the dark. The absorbance was measured at 593 nm. The results were calculated using a standard curve (R^2^ = 0.9982) which was built by dissolving trolox in methanol at different concentrations (15.6, 62.5, 125, 250, 500, and 1000 µM) and expressed as mM of trolox equivalents per g (mM TE/g).

#### 2.4.4. Encapsulation Productivity (EP)

The EP for TPC, TFC and antioxidant capacity (FRAP) was determined using the following equations [15]:

(1) $$EP_{TPC}=\frac{{\mathrm{TPC}}_{c}}{{\mathrm{TPC}}_{\mathrm{all}}}\times \;100$$

(2) $$EP_{TFC}=\frac{{\mathrm{TFC}}_{c}}{{\mathrm{TFC}}_{\mathrm{all}}}\;\times \;100\;$$

(3) $$\;EP_{FRAP}=\frac{{\mathrm{FRAP}}_{c}}{{\mathrm{FRAP}}_{\mathrm{all}}}\;\times \;100\;$$

In the above equations, TPC_c_, TFC_c_ and FRAP_c_ are the amount of TPC, TFC and FRAP respectively in the microparticles, while TPC_all_, TFC_all_ and FRAP_all_ are the amount of TPC, TFC and FRAP respectively in the extracts used for encapsulation.

#### 2.4.5. Moisture Content

The moisture content was determined according to Paini et al. [9]. Powder was dried at 105 °C in an oven until it reached a constant weight. The moisture content was calculated based on the weight loss between before and after drying.

#### 2.4.6. Water Activity (a_w_)

The water activity (a_w_) was determined using a water activity meter (Decagon Devices, Inc., Pullman, WA) according to Vuong et al. [29].

#### 2.4.7. X-ray Diffraction (XRD) Analysis

The XRD analysis was performed using X-ray diffractometer (PANanalytical, X’pert PRO Multi-purpose X-ray diffractometer, Almelo, The Netherland). The radiation was generated at 40 mA and 40 kV. The scattering angle of 2θ from 10° to 99° was measured at the step size of 0.013.

#### 2.4.8. Morphology of Particles by Scanning Electron Microscopy (SEM)

The morphology of the microparticles was determined using a Phillips XL 30 microscope. The Phillips XL 30 microscope was operated at a voltage of 5 kV. A small amount of powder was fixed onto an aluminum specimen holder with double-side tape and covered with gold.

### 2.5. Statistical Analysis

One-way analysis of variance (ANOVA) was performed using SPSS statistical software (version 23, IBM, Crop., NY, USA) at *P* < 0.05. The means were compared with Duncan’s test at *P* < 0.05. The results were expressed as the mean ± standard deviation. Each experiment was conducted in triplicate.

## 3. Results and Discussion

### 3.1. Polyphenol Content and Antioxidant Capacity of the Microparticles

The TPC, TFC, and FRAP values of the lemon by-product aqueous extracts before and after encapsulation can be seen in Table 3. The lemon by-product aqueous extracts before encapsulation had higher TPC, TFC and FRAP values (2.22 ± 0.14 mg GAE/g d.b., 0.58 ± 0.02 mg CE/g d.b. and 5.44 ± 0.10 mM TE/g, respectively) compared to the powders produced either by spray or freeze-drying. During freeze-drying, polyphenol degradation may have occurred due to freezing and dehydration stresses which may have been generated during the encapsulation process, as well as the grinding after lyophilization [30]. During the grinding of the lyophilized material, the surface exposed to oxygen increased, leading to the oxidation of the phenolic compounds and antioxidants. In case of spray-drying, the lower polyphenol and antioxidant capacity values could be attributed to the polyphenol degradation/conversion due to the high inlet temperatures generated during the process [1,12,31]. The TPC and FRAP values of the powders obtained by freeze-drying ranged from 1.30 to 1.66 mg GAE/g d.b., and from 3.36 to 3.70 mM TE/g, respectively. The values obtained by spray-drying ranged from 1.26 to 1.49 mg GAE/g d.b. and from 2.99 to 3.17 mM TE/g, respectively. Ballesteros et al. [6] found that freeze-drying using maltodextrin as coating agent was a more efficient technique than spray-drying for the encapsulation of phenolic compounds extracted from spent coffee grounds. However, in our study, the highest TPC, TFC, and FRAP values obtained by freeze-drying using the mixture of maltodextrin with soybean protein as the coating agent (Table 3). These results could be attributed to the capacity of soybean proteins to interact with various coating agents, including maltodextrin, forming colloidal particles which encapsulate polyphenols Furthermore, due to the high temperature, the degradation of some heat-sensitive phenolic compounds could have produced these results [12,32]. In summary, the use of the polysaccharide-protein proved to be the most efficient coating agent for the encapsulation of lemon pomace aqueous extracts either by freeze-drying or spray-drying.

### 3.2. Encapsulation Productivity (EP)

EP is the ratio (%) between the TPC, TFC, and antioxidant capacity of the extracts used as a core material before encapsulation and the TPC, TFC, and antioxidant capacity of the microparticles obtained after encapsulation either by spray or freeze-drying [15]. The results of the encapsulation productivity are present in Table 3. The highest EP_TPC_, EP_TFC_, and EP_FRAP_ values were obtained by freeze-drying when the mixture of maltodextrin with the soybean protein was used as a coating agent. These results are in agreement with Ballesteros et al. [6] who found that freeze-drying was more efficient for the encapsulation of phenolic compounds extracted from coffee grounds using maltodextrin as a coating agent. For the particles produced by spray-drying, the mixture of maltodextrin with the soybean protein resulted in higher EP_TPC_ and EP_FRAP_ values than those obtained by 100% maltodextrin. However, as expected, the coating agent had no effect on the EP_TFC_ values of the microparticles obtained by spray-drying. The efficiency of coating agents to encapsulate polyphenols is associated to their solubility in dispersion, structure, and capacity to form films [15]. The enhancement of encapsulation productivity with the addition of the soybean protein into maltodextrin was probably due to the interaction of the protein with the maltodextrin and the formation of complexes with interfacial and amphiphilic properties [15]. These results suggest that the encapsulation productivity of lemon by-product aqueous extracts enriched with polyphenols is affected by both the coating agent and the encapsulation technique. Freeze-drying was found to be a more efficient method than spray-drying for the retention of lemon by-product polyphenols and antioxidants.

### 3.3. Morphology and X-ray Diffraction (XRD) Analysis of the Powders

**Figure 3:**
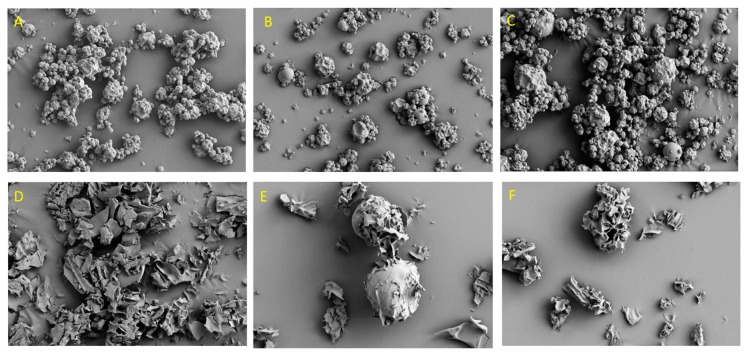
Scanning electron micrographs of the encapsulated lemon pomace aqueous extracts. (**A**) Spray-drying, maltodextrin; (**B**) spray-drying, maltodextrin + soybean protein; (**C**) spray-drying, maltodextrin + ι-carrageenan; (**D**) freeze-drying, maltodextrin; (**E**) freeze-drying, maltodextrin + soybean protein; (**F**) freeze-drying, maltodextrin + ι-carrageenan.

Figure 3 illustrates the morphology of the powders obtained by spray-drying and freeze-drying. The encapsulation technique had a significant impact on the morphology of the microparticles. Spray-drying resulted in the formation of spherical particles of varying diameters, with concavities, irrespective of the choice of the coating agent. Moreover, cracks and pores were not identified on the surfaces of the microparticles (Figure 3A–C). These results show that the formation of concavities is not associated with the coating agent composition, but with the spray-drying conditions. The formation of concavities on the surfaces of the microparticles was attributed to the shrinkage of the particles due to the dramatic loss of moisture after cooling [25,33]. Similar observations reported by Ballesteros et al. [6] who used maltodextrin and gum arabic as coating agents for the encapsulation of phenolic compounds from coffee grounds by using spray-drying. By contrast, the powders produced by freeze-drying had a resembled broken glass or flake-like structure (Figure 3D–F). This structure could be due to the low temperature involved in the freeze-drying process which results in the lack of forces for breaking up the frozen liquid into droplets [34]. Variations in the surface morphology of the powders produced by freeze-drying were observed and could be attributed to the properties of the coating agents. The addition of soybean protein into the maltodextrin resulted in the formation of spherical porous materials (Figure 3E). The spherical microparticles obtained after freeze-drying could be due to the interactions between polyphenols and soybean protein in the feed solution. Polyphenols may bind to the backbone of the protein molecule, leading to the unfolding of the protein chain [35]. The pores correspond to the spaces that were occupied by ice crystals which were removed by sublimation (primary drying) [30]. The loss of porous structure observed in the microparticles, with maltodextrin as a coating agent, could be due to the increased moisture absorption of maltodextrin [36]. These results are in accordance with previous studies, in which the morphology of microparticles produced by spray-drying was compared with those produced by freeze-drying [6,8].

**Figure 4:**
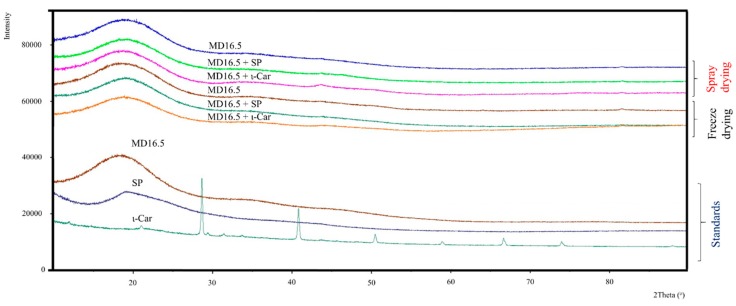
X-ray diffractograms (XRD) for the lemon by-product aqueous extracts encapsulated by freeze-drying and spray-drying, as well as for the pure maltodextrin, soybean protein, and ι-carrageenan. MD: Maltodextrin 16.5–19.0 DE; SP: Soybean protein; ι-Car: ι-carrageenan.

Figure 4 illustrates the XRD patterns for lemon by-product aqueous extracts encapsulated in the different coating agents (maltodextrin, soybean protein, and ι-carrageenan) using spray-drying and freeze-drying. The XRD of the samples revealed a low degree of crystallinity since only one broad pick around 2θ = 18° associating with many noises was detected. The results suggest that neither the encapsulation technique nor the composition of the coating agent influenced the crystallinity of the samples. Similar results were also reported by Tao et al. [15] who showed that the powders of the blueberry anthocyanin extracts produced by freeze-drying using mixtures of various coating agents, including maltodextrin, β-cyclodextrin, whey protein isolate, and gum arabic, were in the amorphous phase, irrespective of the formulations. Similarly, Ballesteros et al. [6] found a very low degree of crystallinity for the powders of coffee ground phenolic extracts produced by freeze-drying and spray-drying using maltodextrin, gum arabic, and their mixture as encapsulation agents.

### 3.4. Moisture Content and Water Activity of the Powders

**Figure 5:**
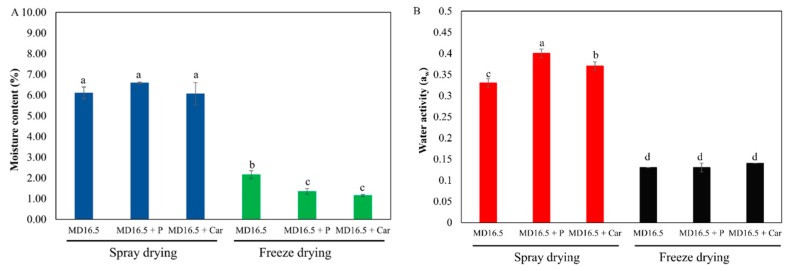
(**A**) Moisture content (%) of the powders produced by spray-drying and freeze-drying, (**B**) water activity (a_w_) of the powders produced by spray-drying and freeze-drying. MD: Maltodextrin 16.5–19.0 DE; P: Soybean protein; Car: ι-carrageenan; Bars followed by different letters are significantly different at *P* < 0.05, according to the Duncan’s test.

Moisture content and water activity are two crucial parameters affecting powder quality since both have an effect on powder shelf life [7]. Water activity (a_w_) represents the availability of free water in a food system which is responsible for biochemical reactions, whereas moisture content represents the water composition of a system [7]. The moisture content of the microparticles produced by spray-drying (varied from 6.06% to 6.60%) was significantly higher than those produced by freeze-drying (varied from 1.15% to 2.15%) (Figure 5A). These results are in accordance with Ramírez et al. [37] who reported lower moisture content in the microparticles produced by freeze-drying compared to those produced by spray-drying. However, Kuck and Noreña [8] reported lower moisture content in the powders obtained by spray-drying compared to those obtained by freeze-drying. These differences could be attributed to the lower inlet temperature (125 °C) used in our study. Higher inlet temperatures during spray-drying have been shown to promote moisture evaporation by enhancing the heat transfer between air and droplets [9]. Similarly, the microparticles produced by spray-drying had significantly higher water activity (varied from 0.33 to 0.40) than those produced by freeze-drying (varied from 0.13 to 0.14) (Figure 5B). Powders obtained by both spray-drying and freeze-drying could be considered micrologically and enzymatically stable since their water activity was lower than 0.60 [7,38]. These results are in contrast with those reported by Kuck and Noreña [8] who found that the grape skin extracts encapsulated by spray-drying using gum arabic, polydextrose, and partially hydrolyzed guar gum as encapsulating agents, had lower water activity than those encapsulated by freeze-drying. These differences could be attributed to the different encapsulating conditions, as well as to the different compositions of the coating agents applied by both studies. In summary, the powders produced by freeze-drying had significantly lower moisture content and water activity compared to those produced by spray-drying.

## 4. Conclusions

Citrus aqueous extracts, due to their polyphenol content and antioxidant capacity, could be utilized by food industry as preservatives. For prolonging their storage life, preserving their beneficial properties, and removing some undesirable odors, the bioactive compounds contained in citrus aqueous extracts should be encapsulated. However, undesirable encapsulation conditions and wall materials may lead to the degradation of some bioactive compounds. Therefore, the encapsulation technique and the wall material must be carefully selected for retaining the beneficial properties of the extracts. Both the composition of the coating agent and the encapsulation technique were found to significantly affect the encapsulation of phenolic compounds and antioxidants of lemon by-product aqueous extracts. In general, freeze-drying and the mixture of maltodextrin (16.5–19.0 DE) with soybean protein were found to be the most efficient for the production of powders with the highest TPC, TFC, and antioxidant capacity (FRAP). Scanning electron microscopy revealed that spray-drying resulted in the formation of spherical microparticles of different sizes, with concavities, regardless of the type of coating agent. On the other hand, freeze-drying resulted in powders of amorphous glassy shapes. However, the mixture of maltodextrin with soybean protein as the coating agent resulted in the formation of spherical porous materials during freeze-drying. Comparing the two encapsulation techniques, freeze-drying resulted in powders with lower moisture content and water activity compared to those produced by spray-drying. Although freeze-drying proved to be a more efficient technique for the encapsulation of lemon by-product aqueous extracts than spray-drying, it is considered an expensive technique due to the prolonged processing times required. Therefore, more studies should be conducted to investigate the efficiency of different encapsulation techniques using different coating agents.

## Figures and Tables

**Table 1:** Summary of studies on the encapsulation of citrus pomace extracts.

Citrus Species	Coating Agents	Encapsulation Technique	Refs
Orange peels	Gelatin and Gum arabic	Coacervation	[16]
Orange peels	Whey protein isolate	Spray-drying	[17]
Orange peels	Whey protein isolate	Spray-drying	[18,19]
Citrus peels	Whey protein isolate	Spray-drying	[20]
Orange peels	N.M.	Spray-drying	[21]

N.M.: Not mentioned.

**Table 2:** Ratios of coating agents (g/g) employed for the encapsulation of lemon by-product extracts by spray or freeze-drying.

MD (16.5–19.0 DE)	Soybean Protein	ι-Carrageenan
1	0	0
5	1	0
9	0	1

**MD**: maltodextrin; DE: dextrose equivalent.

**Table 3:** Total phenolic content (TPC) (mg GAE/g d.b.), total flavonoid content (TFC) (mg CE/g d.b.), ferric reducing ability of plasma (FRAP) (mM TE/g), encapsulation productivity (EP %) for TPC, TFC and FRAP of the microparticles. The results were expressed as mean ± standard deviation (*n* = 3).

Method	Coating Agents	TPC	TFC	FRAP	EP_TPC_	EP_TFC_	EP_FRAP_
Spray-drying	MD	1.26 ± 0.03 ^d^	0.34 ± 0.01 ^c^	2.99 ± 0.02 ^e^	56.52 ± 1.57 ^d^	58.14 ± 1.51 ^b^	54.84 ± 0.35 ^d^
	MD + SP	1.49 ± 0.03 ^c^	0.34 ± 0.01 ^c^	3.17 ± 0.07 ^d^	66.97 ± 1.16 ^b^	58.14 ± 0.75 ^b^	58.23 ± 1.33 ^c^
	MD + ι-Car	1.26 ± 0.02 ^d^	0.34 ± 0.01 ^c^	3.10 ± 0.04 ^d,e^	56.46 ± 0.84 ^d^	58.67 ± 1.51 ^b^	56.90 ± 0.73 ^c^
Freeze-drying	MD	1.41 ± 0.10 ^c,d^	0.33 ± 0.00 ^c^	3.36 ± 0.02 ^c^	63.45 ± 4.78 ^b,c^	56.53 ± 0.01 ^b^	61.74 ± 0.41 ^b^
	MD + SP	1.66 ± 0.02 ^b^	0.43 ± 0.02 ^b^	3.70 ± 0.05 ^b^	74.84 ± 1.05 ^a^	74.40 ± 2.64 ^a^	68.02 ± 0.86 ^a^
	MD + ι-Car	1.30 ± 0.01 ^d^	0.33 ± 0.02 ^c^	3.43 ± 0.04 ^c^	58.46 ± 0.32 ^c,d^	57.07 ± 3.02 ^b^	63.07 ± 0.76 ^b^
	Extract	2.22 ± 0.14 ^a,^*	0.58 ± 0.02 ^a^	5.44 ± 0.10 ^a^			

* Values followed by different letters in the same column are significantly different at *P* < 0.05, according to the Duncan’s test. MD: Maltodextrin 16.5–19.0 DE; SP: Soybean protein; ι-Car: ι-carrageenan.
